# Validation of TMMS-24 in Three Spanish-Speaking Countries: Argentina, Ecuador, and Spain

**DOI:** 10.3390/ijerph18189753

**Published:** 2021-09-16

**Authors:** Ana Belén Górriz, Edgardo Etchezahar, Diego E. Pinilla-Rodríguez, María del Carmen Giménez-Espert, Ana Soto-Rubio

**Affiliations:** 1 Department of Developmental Psychology, Universitat Jaume I, 12071 Castellón, Spain; gorriz@uji.es; 2 Faculty of Psychology, Universidad Nacional de Buenos Aires, Buenos Aires 1417, Argentina; edgardoetchezahar@psi.uba.ar; 3 Faculty of Political and Administrative Sciences, Universidad Nacional de Chimborazo, Chimborazo 060110, Ecuador; dpinilla@unach.edu.ec; 4 Department of Nursing, Faculty of Nursing and Chiropody, University of Valencia, 46010 Valencia, Spain; 5 Department of Personality, Assessment and Psychological Treatments, Faculty of Psychology, University of Valencia, 46010 Valencia, Spain; ana.soto@uv.es

**Keywords:** validation, emotional intelligence, intergroup relations, mood, TMMS-24

## Abstract

Emotional intelligence (EI) is a fundamental skill related to different aspects of human life, such as psychological well-being or mood states. The present study has a triple objective: first, to explore the psychometric properties of the TMMS-24 in three Spanish-speaking countries (Argentina, Ecuador, and Spain); second, to examine the relation of EI with mood and avoidance of responsibility; and finally, to analyse the influence of sex, age and national differences on EI. The relevance of this study is given by the need for tools to assess EI in different cultures. A sample of 1048 adults (*M*_age_ = 21.11 years, *SD* = 5.84; 52.3% male) was selected by convenience sampling. The psychometric properties of the TMMS-24 were adequate, and the Spanish sample showed lower levels of EI than the Argentinian and Ecuadorian ones. EI was associated with mood and the avoidance of responsibility, with higher levels in women in all cases. Regarding the national and sex-specific differences, the Spanish sample showed significant differences in attention and repair, with men exhibiting higher scores in attention and women having higher scores in repair. In the Argentinean sample, no significant differences were found, and in the Ecuadorian one, women presented higher scores in attention than men. The TMMS-24 can be considered a useful, practical tool to assess EI in adults in different cultures and with different languages.

## 1. Introduction

Emotional intelligence (EI) is a construct that refers to the processes of recognition, understanding and regulation of our emotional states and of others, as well as their implementation to solve problems and regulate behaviour [1]. It has aroused great interest among researchers, being widely studied in different areas such as physical and mental health [2,3,4,5,6], education [7], sports, work, and organisations [8,9,10,11,12], among others, proving its importance and impact in all them [13].

EI is related to aspects such as psychological well-being or mood states [14,15], school adjustment [16,17], and interpersonal relationships [18,19,20]. Moods play a very important role in daily life and are affected by several personal variables, such as EI, and they are essential for personal and social adjustment, as well as problem-solving [21]. Moods have been defined as diffuse affective states that appear without any specific reason [22]. There are studies that indicate that people with high EI show lower negative moods than people with low EI [13,23,24,25], and they also recover better from a negative mood than people with low EI [25].

EI is also related to mental health. Higher EI is associated with a healthier emotional and physiological response and less use of avoidance, ignoring, or distraction strategies to cope with stressful situations [26]. Likewise, it has been related to adequate coping with stressful situations [27,28,29], motivation [30,31], problem-solving [7], self-esteem [32], adaptive use of emotions [3], and less disruptive behaviour [16,33]. These aspects are fundamental because the fulfilment of responsibilities is one of the most important factors for individuals to achieve success [34]. Therefore, a responsible individual is a person who respects himself and others, takes care of his or her own affairs, is aware of his or her own affairs, and takes responsibility for everything related to it [35]. In this context, responsibility avoidance is understood as “those strategies that are used to avoid responsibility for their inappropriate behaviours. Those strategies include lying, cheating, and a variety of incorrect attributions” [36] (p. 549). Responsibility avoidance would be denying the personal blame for disruptive behaviours by using biased and distorted strategies. Sutton, Reeves, and Keogh [37] found via factor analysis that responsibility avoidance is comprised of three factors: personal justification (reflects a tendency to justify disruptive behaviour in terms of difficult life experiences), shifting blame (refers to the core of avoidance of responsibility, including elements that indicate the transfer of responsibility to others), and denial/lack of remorse (a denial of personal responsibility for bad behaviour and a lack of remorse for the actions).

In general, high EI is correlated with more adaptative ways of coping with health threats, better manage daily life problems [38], greater resilience [39], emotional regulation [40] and well-being [41], and less avoidance of responsibility [42]. Moreover, research highlights sex and cultural differences in the perception, expression, and regulation of emotions [43]. Emotional experiences and behaviours that fit a particular culture are likely to be reinforced by that culture. Human societies tend to be either individualistic, endorsing values such as emotional expression and the right to free choice, or collectivistic, placing less emphasis on emotional expression as essential to well-being [44]. Previous studies have found differences in EI between individualistic and collectivistic societies [45]. Moreover, women have better social skills and are better at understanding the emotions of others in comparison to men [46].

Despite these important aspects, there are no studies that have analysed EI by comparing different Spanish-speaking countries. In this sense, there is a need of designing and validating EI assessment tools with appropriate psychometric properties in different contexts [47]. For all of these reasons, this study aims to adapt and validate the TMMS-24 in the adult population of three Spanish-speaking countries (Argentina, Ecuador, and Spain). In addition, the relationship with other variables such as moods and avoidance of responsibility is examined. Finally, the possible influence of sex, age, and culture on EI is analysed.

## 2. Materials and Methods

### 2.1. Participants

A cross-sectional study was conducted in a sample of 1048 adults from a middle-class socioeconomic background, aged 18 to 59 years (*M_age_* = 21.11 years, *SD* = 5.84; 52.3% male). The sample was selected by convenience sampling from urban areas of three Spanish-speaking countries (Spain, Argentina, and Ecuador). Regarding the different samples, 464 were from Spain (363 male, aged between 18 and 55, *M_age_* = 20.63 years, *SD* = 3.92), 221 were from Argentina (50 male, aged between 18 and 59, *M_age_* = 26.63 years, *SD* = 9.72), and 363 were from Ecuador (128 male, aged between 18 and 50, *M_age_* = 20.66 years, *SD* = 2.18). Data were collected in universities in urban areas of the city of Buenos Aires (Argentina), the province of Chimborazo (Ecuador), and the Valencian community (Spain). Data from the indigenous population usually located in rural areas and periphery of large cities were not collected (Buenos Aires: indigenous population 1.9% (National Institute of Statistics and Census of Argentina, 2010); Chimborazo: indigenous population 17.1% (National Institute of Statistics and Census of Ecuador, 2010); Valencian community; university students nationality Latin America and Caribbean 1.19% (Statistics of students academic year 2017/2018. Ministry of Universities, Spain)). The present study was authorised by the University of Valencia Human Research Ethics Committee H1529396558647. All participants received detailed information about the aim and procedures and were informed about confidentiality. Informed consent was obtained from all participants.

### 2.2. Instruments

Trait Meta-Mood Scale [12,48,49] or TMMS [49] based on Mayer and Salovey model (1997) [50]. This scale assesses people′s beliefs about their own emotional intelligence. Specifically, on their ability to attend, clarify and repair their emotional states. In its extensive version, the scale consists of 48 items, although the most used is the 24 items version, which presents adequate internal consistency and convergent validity according to various researches [49,51]. The TMMS-24 is a self-report measure designed to assess individuals′ beliefs regarding their own emotional abilities. This scale consists of a 24-item Likert-type scale on which participants are required to rate the extent to which they agree with each item (1 = Strongly disagree and 5 = Strongly agree). These items are grouped on three key aspects of perceived EI, specifically attention, clarity, and repair. Attention conveys the extent to which individuals tend to observe and consider their feelings and moods. Clarity makes reference to the understanding of one′s own emotional states. Repair refers to individuals′ beliefs concerning their ability to regulate their feelings. The authors reported adequate psychometric characteristics, and these features have been replicated in this study (attention: α = 0.92; clarity: α = 0.84; repair: α = 0.84).

The Mood Questionnaire (MOOD) [52], adapted to Spanish by Górriz et al. [53]. This questionnaire comprises four mood scales: happiness, anger, sadness, and fear. The participants are instructed to answer each item on a three-point response scale (1 = Never, 2 = Sometimes, 3 = Often). In all dimensions, higher scores indicate a greater presence of the mood state. The different dimensions presented adequate alpha values in previous studies as well as in this research (sadness: α = 0.69; fear: α = 0.69; anger: α = 0.78; happiness: α = 0.76).

The Avoidance of Responsibility Scale (ARS) [37]. It consists of 28 Likert-type items grouped in the three factors: personal justification, shifting blame, and denial/lack of remorse. According to the authors [37], this scale presents adequate psychometric properties, which were also replicated in the present study with a total alpha of 0.70 and values that range between 0.62 and 0.80 on each dimension.

### 2.3. Procedure

The self-report scales were completed in the classroom by the participants. The assessment lasted around 30 min, and it was anonymous and voluntary. Data were collected from college students from four universities located in three Spanish-speaking countries: Spain, Argentina, and Ecuador. The data collection process occurred from September to December of 2018.

### 2.4. Data Analysis

The statistical analysis was conducted using SPSS 22.0, EQS 6.3, and SBDIFF. First, descriptive statistics for every item were calculated, followed by the analysis of empirical evidence for the reliability and validity of the scale on each population, and then by a multi-group analysis using structural equation models (SEM). Later, the factorial invariance was tested. Afterward, the link with MOOD and ARS was examined. Finally, the influence of sex and age on the TMMS-24 was analysed.

## 3. Results

### 3.1. Analyses of Items and Reliability

The 24 items that compose the TMMS-24 scale were analysed. According to the criteria of reliability, six items (5,6,13,15,17,23) had to be eliminated in order to improve Cronbach’s alpha dimension. The results presented below refer to this 18 items scale.

Table 1 shows the final wording of the 18 items and, for each item, its mean, standard deviation, item-total correlation, and Cronbach′s alpha if the element is deleted in the whole sample as well as in the three different countries.

In general, all items seem to make an adequate contribution to the scale as a whole. The reliability of the different dimensions is adequate for the whole sample considering the Cronbach’s alpha (attention: α = 0.88; clarity: α = 0.87; repair: α = 0.87), as well as when considering the Spanish sample (attention: α = 0.90; clarity: α = 0.87; repair: α = 0.87), the Argentinean sample (attention: α = 0.83; clarity: α = 0.86; repair: α = 0.85) and the Ecuadorian sample (attention: α = 0.86; clarity: α = 0.86; repair: α = 0.83). When comparing data from the three samples reliability seems to be higher in the Spanish one. All the results are also adequate when considering the composite reliability coefficient (CRC). These results do not improve when removing any of the items.

### 3.2. Validity Analysis

After analysing the reliability of the scale, the next step was to study its validity. First, the adequacy of the data was analysed by the Kaiser-Meyer-Olkin test (KMO); >0.80 is an adequate value [54]. The results showed KMO values of 0.90 for the whole sample, 0.89 for the Spanish sample, 0.83 for the Argentinian sample, and 0.86 for the Ecuadorian sample. Bartlett’s test of sphericity was adequate (*p* < 0.001) in all samples. Mean component analyses were used to calculate an exploratory factor analysis (EFA), with eigenvalues greater than 1 criterion. This criterion was confirmed by both the analysis of the screen test and by a parallel analysis (PA) [55]. The final model was composed of three factors as the original structure [51], which accounted for 61.71% from the whole sample; 63.54% from the Spanish sample; 58.30% from the Argentinian sample, and 58.22% from the Ecuadorian sample.

Then, several confirmatory factor analyses (CFA) were calculated. First, a CFA considering the whole sample, then a CFA on each of the samples (Spain, Argentina, and Ecuador), and finally, the factorial invariance was calculated. In all cases, ML (maximum-likelihood) estimation was used with the Satorra-Bentler robust correction to control for possible non-normality of the data.

Based on the significance of the χ^2^ statistic (<0.01), an adequate model fit cannot be ensured in any of the cases. However, given that this analysis is very sensitive to the sample size, other goodness-of-fit indices were applied, such as the Non-Normed Fit Index (NNFI), the Comparative Fit Index (CFI), the Incremental and the McDonald′s Fit Indices (IFI and MFI, respectively), with values above 0.90 indicating good fit, and the Root Mean-Square Error of Approximation (RMSEA), in which an adequate fit would score equal to or below 0.08 [56]. Table 2 presents a summary showing these indicators for each of the CFA.

The results found seem to justify the internal validity of the instrument in the three countries. In order to analyse the factorial invariance, changes in model fit with the inclusion of constraints were tested by a significance test on the difference between Satorra-Bentler scaled chi-square test using SBDIFF [57,58]. The results (Table 2) allow us to assume equal form invariance and equal factor loadings invariance considering the three countries.

The adequacy of the CFA of the proposed 18 items model showed better fit with a chi-squared value (S-Bχ2 = 571.51; df = 132), and the chi-squared value and its degrees of freedom (χ2 = 690.74; df = 132). The RMSEA was 0.06, which agrees with the minimal acceptable fit criteria (≤0.08). Similarly, the remaining indices also showed a better fit: NNFI = 0.93; CFI = 0.94; and IFI = 0.94.

Furthermore, the convergent validity of the scale on the basis of the results obtained in the CFA was tested. The 18 items that make up the TMMS instrument were significantly and strongly correlated with the latent variable they assumed to measure; in all cases, the *t* values were above 3.291, and they failed to improve when new loads were included. Finally, discriminant validity was evaluated by means of the average variance extracted test (AVE) [59]. To determine the existence of discriminant validity, the AVE square root must be higher than the correlation among the pairs of factors or dimensions considered [59]. The results displayed in Table 3 suggest an acceptable discriminant validity.

Following the analyses as suggested by the literature, the relations of the construct with other variables were examined in order to determine the criterion/nomological validity of this instrument [38,52,60,61]. Hence, Pearson correlation coefficients were calculated for the TMMS-24, MOOD, ARS, and age dimensions (Table 4), and the prediction of ARS was analysed by the emotional component in all samples.

Regarding correlations between TMMS-24 and MOOD, considering the whole sample, clarity and repair correlate negatively with fear and anger and positively with happiness, while attention correlates positively with fear and sadness. Finally, sadness correlates negatively with repair. These relations were also observed in the other samples, with the exception of fear and TMMS-24 dimensions in Argentina, attention and fear in Ecuador, and clarity and anger in Spain, Argentina, and Ecuador, which do not present significative correlations.

With regard to the relations with the ARS instrument, all correlations were low and negative, with the exceptions of attention and personal justification, which correlated positively, and attention and denial/lack of remorse, and repair and personal justification, which did not correlate. These correlations were also observed in Spain, Ecuador, and Argentina, with the exception of the correlation between attention and ARS dimensions. In addition, and in the case of Argentina, personal justification does not correlate with any of the TMMS-24 dimensions. Lastly, in all cases in Spain, higher correlations are to be found.

### 3.3. Prediction of ARS with TMMS-24 and MOOD

The prediction of the Avoidance of Responsibility Scale (ARS) by the emotional component was analysed in the whole sample as well as in the sub-samples. Hierarchical regression analyses were carried out, with ARS as criterion variable and TMMS-24 and MOOD as predictor variables. As a first step, all of the subscales of the TMMS-24 were included and subsequently the dimensions of MOOD. Although the ARS considers three dimensions, only the information regarding personal justification is presented since the prediction of the other dimensions is lower than 10%.

Considering the whole sample, attention (β = 0.11; *p* < 0.001), clarity (β = 0.12; *p* < 0.001) and repair (β = −00.04; *p* < 0.001) predict 2% of the variance of personal justification. With the addition of MOOD, the model improved significantly, with R^2^ increased by 0.11 and the total variance explained rising up to 13%. Regarding MOOD, fear (β = 0.13; *p* < 0.001), and sadness (β = 0.20; *p* < 0.001) positively predict personal justification.

When analysing the samples from Spain, Argentina and Ecuador separately, the following predictions were observed. In Spain, attention (β = −0.16; *p* < 0.001) and clarity (β = −0.17; *p* < 0.001) predict 3% of the variance of personal justification, and the addition of MOOD (fear: β = 0.18; *p* < 0.001) improved the model significantly, with R^2^ increased by 0.10, and the total variance explained rising up to 12%.

In Argentina, the TMMS-24 dimensions (attention β = 0.13; *p* < 0.05; clarity β = −0.24; *p* < 0.001) predict 5% of the personal justification variance, with the addition of MOOD (fear β = −0.24; *p* < 0.001 and sadness β = −0.25; *p* < 0.001) the model improved significantly, with R^2^ increased by 0.23, and the total variance explained rising up to 25%. Finally, in Ecuador, sadness (β = 0.12; *p* < 0.001) predicts 12% of variance of personal justification.

### 3.4. Correlations

The next step was the analysis of the Pearson correlations between the instrument dimensions (Table 3) using the whole sample and data from the three countries. There were statistically significant positive low correlations among all dimensions (*p* < 0.01). Results were almost the same when comparing the different countries.

### 3.5. Differences in TMMS-24 Considering the Three Countries

In order to analyse the differences in EI dimensions considering the three countries, an ANOVA with Tukey posteriori contrast was performed. Based on the results obtained, there are significant differences in all dimensions considering the three countries. Specifically, in Spain, attention levels are significantly lower (*p* < 0.01) than in Argentina and Ecuador, while levels in Argentina are statistically higher than in Ecuador. Likewise, the Spanish sample presents significantly lower levels (*p* < 0.001) in clarity than the Argentinian and Ecuadorian samples. Finally, in terms of repair, the levels are significantly lower (*p* < 0.001) in Spain than in Argentina or Ecuador and lower in Argentina than in Ecuador.

### 3.6. Influence of Sex and Age

Lastly, the effects of sex and age on the differences in TMMS-24 were analysed. First, the sex-specific mean scores for the different samples were compared by a t-test (Table 5); then, the factorial invariance of the instrument according to sex was tested (Table 2).

The sex-specific differences were statistically significant for the whole sample in attention (*t* = −3.72; *p* < 0.01; Male: *M* = 21.86; Female: *M* = 23.04; Cohen’s *d* = 0.18), clarity (*t* = −2.33; *p* < 0.05; Male: *M* = 20.18; Female: *M* = 20.91; Cohen’s *d* = 0.12) and repair (*t* = −2.12; *p* < 0.05; Male: *M* = 19.49; Female: *M* = 21.61; Cohen’s *d* = 0.12). As it can be seen, in all cases women presented higher levels than man.

Considering the different countries, in Spain significative differences were observed in attention (*t* = 3.23; *p* < 0.001; Male: *M* = 21.59; Female: *M* = 19.69; Cohen’s *d* = 0.31) and repair (*t* = −2.03; *p* < 0.05; Male: *M* = 18.29; Female: *M* = 19.55; Cohen’s *d* = 0.54) with higher values of men in attention and higher of women in repair. In the Argentinean sample, we do not observe significative differences, and in Ecuador we observe differences in attention (*t* = −3.84; Male: *M* = 21.50; Female: *M* = 23.60) with higher values of women.

Regarding the factorial invariance of the instrument according to sex, the results on S-B scaled difference χ^2^ = 14.36, *df* = 15; (*p* = 0.50) allow to assume both equal form invariance and equal factor loadings invariance considering the sex.

Finally, regarding age, significant low positive correlations were observed with all TMMS-24 dimensions in the whole sample. When considering the different countries, only attention and repair presented significantly low positive correlations.

## 4. Discussion

One of the aims of this study was to test the psychometric properties of the TMMS-24 in three Spanish-speaking countries (Argentina, Ecuador, and Spain). According to the criteria of validity and reliability, six items were removed from the original scale in order to have an instrument with adequate psychometric properties for the three countries studied. Thus, an 18-item scale was used to evaluate An analysis of the 18 items showed that all of the items seemed to make an adequate contribution to the scale overall. The reliability considering the Cronbach’s alpha and the CRC of the three dimensions are adequate, higher than 0.80 in all cases, over the minimum value (>0.70) suggested in the literature [62].

The next step was to test the validity of the scale. The resulting model (EFA and CFA) consisted of three dimensions, as well the original structure, and showed the internal validity of the instrument in the three countries (Spain, Argentina, and Ecuador). The results on S-B scaled difference allow us to assume equal form invariance and equal factor loadings invariance considering the three countries. In addition, the scale presents convergent and discriminant validity. These psychometric properties seem to justify the use of the instrument in the three Spanish-speaking countries (Argentina, Ecuador, and Spain). Furthermore, this instrument has been used in different cultures and countries such as Spain [63,64], Mexico [65], Turkey [66], or China [67]. However, stronger validity evidence supporting scores in TMMS-24 contexts outside Spain is needed [65,68]. For this reason, this research evaluates the factor structure of the instrument in three Spanish-speaking countries in the same study.

Regarding the relations among TMMS-24, MOOD, and ARS, considering the results obtained, the influence of the emotional component when experiencing a conflict situation has been considerable. The adults who participated in this study seemed to justify their failure to accept responsibility for their actions based on the negative mood that they experienced. For example, fear or sadness affected personal and social adjustment [37,61], whereas EI appears to promote the acceptance of responsibility [3,26,37,43]. These results call upon reflecting on a possible intervention focused on minimising these negative moods and developing EI to promote acceptance of responsibility for one′s actions [69].

Another aim of this study was to analyse the effect of national differences in EI. It seems that in Spain, attention levels are significantly lower than in Argentina and Ecuador. Furthermore, higher values were observed in Argentina when comparing with Ecuador. Regarding clarity, values are significantly lower in Spain than in Argentina and Ecuador. Finally, concerning repair, levels seem to be significantly lower in Spain when comparing with Argentina and Ecuador and lower in Argentina when compared with Ecuador. These results could be explained by previous research suggesting that there are cultural differences in the perception, expression, and regulation of emotions depending on different cultures, and on whereas the society is more individualistic or collectivistic. Despite both culture and people can present traits of individualism and collectivism at the same time, and that they are neither mutually exclusive nor universally applicable [70], there are cultural differences in the perception, expression, and regulation of emotions in individualistic traits such as the Spanish culture [71] than in collectivistic cultures such as Argentina and Ecuador [72]. In collectivist cultures, there is an explicit expression of positive emotions, while in individualistic cultures, there is a more subjective experience of these emotions, with more facial and bodily expression [72]. In addition, the literature shows studies that explain these cultural differences through middle-class socioeconomic background: the higher the income and higher the socioeconomic level, the greater the attention and emotional clarity and the better the use of emotional regulation strategies [73]. In the present study, the socioeconomic background cannot explain these differences in EI (GDP; gross domestic product per capita 2018; Ecuador 5.350€; Argentina 9.931€ and Spain 25.770€) [74].

The effects of age and sex on the differences in TMMS-24 were analysed. The sex-specific differences were statistically significant for the whole sample in all dimensions, with higher levels in women than in men in all cases. These results are consistent with previous research [46,75]. Regarding age, significant low positive correlations were observed with all TMMS-24 dimensions in the whole sample. When considering the different countries, only attention and repair present significant low positive correlations. Finally, factorial invariance of the instrument according to sex was proved.

It would be interesting to extend this research to other populations worldwide and to study the temporal stability of the data from a longitudinal perspective. It could also be interesting to use other external measures, including the participant′s cultural background. Nevertheless, it is necessary to emphasise the novel dimension of this study because it is the first study of the psychometric properties of the TMMS-24 scale in three Spanish-speaking countries.

## 5. Conclusions

Emotional intelligence is a fundamental skill related to different aspects of human life, such as psychological well-being or mood states. The present research provides initial findings to suggest that the TMMS-24 questionnaire can be considered a useful, practical tool to evaluate EI in adults in different cultures and with different languages. This study emphasises the importance of minimising negative moods and developing EI to promote acceptance of responsibility for one′s actions. It also highlights that, in general, women present higher levels of EI than men. These aspects play a key role when identifying educational needs and developing intervention programmes.

## Figures and Tables

**Table 1:** Item analysis: Mean (*M*), standard deviation (*SD*), item-total correlation (*r_jx_*), and Cronbach’s alpha if item deleted (*α.-x*) in samples.

Items	Whole Sample (*n* = 1048)				Spain (*n* = 463)				Argentina (*n* = 220)				Ecuador (*n* = 363)			
	*M*	*SD*	*r_jx_*	*α.-x*	*M*	*SD*	*r_jx_*	*α.-x*	*M*	*SD*	*r_jx_*	*α.-x*	*M*	*SD*	*r_jx_*	*α.-x*
**Attention**	**α = 0.88; CRC = 0.89; AVE = 0.56**				**α = 0.90; CRC = 0.90; AVE = 0.60**				**α = 0.83; CRC = 0.85; AVE = 0.49**				**α = 0.86; CRC = 0.86; AVE = 0.51**			
1. I pay much attention to my feelings	4.11	0.98	0.66	0.87	3.93	0.95	0.71	0.88	4.45	0.86	0.71	0.88	4.15	1.04	0.60	0.85
2. Usually I care much about what I’m feeling	3.96	1.03	0.75	0.85	3.75	1.02	0.75	0.88	4.32	0.89	0.75	0.88	4.01	1.04	0.73	0.82
3. It is usually a waste of time to think about your emotions	3.62	1.11	0.76	0.85	3.41	1.13	0.79	0.87	3.94	1.07	0.79	0.87	3.71	1.04	0.69	0.83
4. I think it’s worth paying attention to your emotions or mood	3.93	1.00	0.65	0.87	3.80	0.96	0.67	0.89	4.19	1.02	0.67	0.89	3.95	1.00	0.66	0.84
7. I often think about my feelings	3.40	1.11	0.65	0.87	3.12	1.11	0.67	0.89	3.73	1.05	0.67	0.89	3.58	1.05	0.62	0.84
8. I pay a lot of attention to how I feel	3.53	1.12	0.70	0.86	3.25	1.14	0.77	0.87	3.80	1.10	0.77	0.87	3.72	1.02	0.62	0.84
**Clarity**	**α = 0.87; CRC = 0.86; AVE = 0.52**				**α = 0.87; CRC = 0.87; AVE = 0.53**				**α = 0.86; CRC = 0.85; AVE = 0.49**				**α = 0.86; CRC = 0.85; AVE = 0.50**			
9. I am usually very clear about my feelings	3.49	1.17	0.67	0.84	3.29	1.11	0.66	0.85	3.47	1.25	0.70	0.82	3.77	1.13	0.64	0.83
10. I am rarely confused about how I feel	3.41	1.10	0.77	0.82	3.16	1.04	0.76	0.83	3.55	1.19	0.79	0.80	3.67	1.06	0.74	00.81
11. I usually know my feelings about a matter	3.45	1.06	0.72	0.83	3.26	1.03	00.73	0.83	3.48	1.11	0.69	0.82	3.67	1.03	0.69	0.82
12. I can make sense out of my feelings	3.64	1.01	0.57	0.86	3.48	0.96	0.58	0.86	3.95	0.98	0.58	0.84	3.68	1.04	0.55	0.85
14. Always I can tell how I feel	3.21	1.09	0.64	0.85	2.96	1.04	0.64	0.85	3.19	1.13	0.59	0.84	3.54	1.05	0.65	0.83
16. I almost always know exactly how I am feeling	3.52	1.02	0.59	0.85	3.38	1.00	0.62	0.85	3.68	1.00	0.51	0.85	3.62	1.03	0.59	0.84
**Repair**	**α = 0.87; CRC = 0.87; AVE = 0.54**				**α = 0.87; CRC = 0.88; AVE = 0.53**				**α = 0.85; CRC = 0.84; AVE = 0.49**				**α = 0.83; CRC = 0.84; AVE = 0.48**			
18. No matter how badly I feel, I try to think about pleasant things	3.53	1.26	0.76	0.83	3.12	1.22	0.79	0.83	3.51	1.29	0.75	0.80	4.06	1.08	0.65	0.808
19. When I am upset, I think of all the pleasure of life	3.00	1.33	0.72	0.84	2.58	1.28	0.73	0.84	2.94	1.27	0.73	0.81	3.57	1.22	0.60	0.81
20. I try to think good thoughts no matter how badly I feel	3.46	1.27	0.80	0.82	3.08	1.24	0.81	0.83	3.49	1.30	0.74	0.80	3.94	1.13	0.76	0.77
21. If I find myself getting mad, I try to calm myself down	3.44	1.16	0.61	0.86	3.18	1.16	0.63	0.86	3.63	1.18	0.53	0.84	3.65	1.07	0.60	0.81
22. I worry about being in a too good mood	3.76	1.07	0.56	0.86	3.54	1.02	0.57	0.87	3.95	1.00	0.50	0.85	3.93	1.11	0.54	0.82
24. When I am angry I don’t usually let myself feel that way	3.43	1.14	0.56	0.87	3.14	1.05	0.53	0.87	3.55	1.15	0.55	0.84	3.73	1.17	0.50	0.83

**Table 2:** Goodness-of-fit index of the TMMS-24 scale in the three countries.

Model	χ^2^ (*df*)	S-B χ^2^ (*df*)	NNFI	CFI	IFI	RMSEA (95% CI)	S-B Scaled Difference (*df)* (*p*-Value)
Whole sample	1529.89 (249)	1260.60 (249)	0.88	0.90	0.90	0.07 (0.062–0.069)	
Whole Sample reespecified (without 5,6,13,15,17,23)	690.74 (132)	571.51 (132)	0.93	0.94	0.94	0.06 (0.054–0.064)	
España	454.12 (132)	389.72 (132)	0.92	0.93	0.94	0.07 (0.060–0.075)	
Argentina	304.13 (132)	252.74 (132)	0.89	0.90	0.91	0.07 (0.054–0.080)	
Ecuador	332.49 (132)	276.80 (132)	0.91	0.92	0.92	0.058 (0.049–0.068)	
Multi-group Equal Form	1120.99 (402)	942.40 (402)	0.91	0.92	0.92	0.07 (0.059–0.070)	
Multi-group Equal Loading	1181.23 (432)	999.86 (432)	0.91	0.92	0.92	0.06 (0.059–0.069)	56.17_(30)_ (0.13)
Men	523.87 (132)	447.61 (132)	0.92	0.93	0.93	0.07 (0.063–0.077)	
Women	347.94 (132)	281.98 (132)	0.94	0.95	0.95	0.05 (0.042–0.058)	
Multi-group Equal Form	713.83 (267)	599.91 (267)	0.93	0.94	0.94	0.06 (0.056–0.070)	
Multi-group Equal Loading	728.90 (282)	616.45 (282)	0.93	0.94	0.94	0.06 (0.055–0.068)	14.36_(15)_ (0.50)

*p* > 0.001; χ^2^/df; S-B χ^2^/df: adequate ≤ 5; NNFI, CFI, IFI ≥ 0.90; RMSEA ≤ 0.08.

**Table 3:** Correlations between TMMS-24 factors.

		1. Attention	2. Clarity	3. Repair
Whole sample	1	(0.75)		
	2	0.35 **	(0.72)	
	3	0.23 **	0.43 **	(0.73)
Spain	1	(0.78)		
	2	0.36 **	(0.73)	
	3	0.16 *	0.37 **	(0.73)
Argentina	1	(0.70)		
	2	0.23 **	(0.70)	
	3	0.16 **	0.48 **	(0.70)
Ecuador	1	(0.72)		
	2	0.33 **	(0.70)	
	3	0.25 **	0.35 **	(0.69)

* *p* < 0.05; ** *p* < 0.01; Note: AVE square root on the diagonal.

**Table 4:** Correlations of the TMMS-24 instrument with MOOD and ARS.

		MOOD				ARS			AGE
		Fear	Happiness	Anger	Sadness	PJ	SB	LR	
Whole Sample	Attention	0.12 **	−0.00	0.12	0.15 **	0.11 **	−0.02 **	0.11	0.09 **
	Clarity	−0.11 **	0.18 **	−0.08 **	−0.17	−0.14 **	−0.04 **	−0.00 **	0.13 **
	Repair	−0.12 **	0.28 **	−0.18 **	−0.20 **	−0.12	−0.06 **	−0.02 **	0.12 **
Spain	Attention	0.15 **	−0.01	0.19 **	0.24 **	0.14 **	−0.02	0.13	−0.02 **
	Clarity	−0.13 **	0.16 **	−0.05	−0.12 **	−0.17 **	−0.09 **	−0.05 **	0.07
	Repair	−0.17 **	0.33 **	−0.19 **	−0.26	−0.15 **	−0.19 **	−0.01 **	0.03 **
Argentina	Attention	0.05	0.01	0.04 **	0.08 *	0.11	−0.01	0.09	0.00 **
	Clarity	−0.10	0.30 **	−0.25	−0.29 **	−0.28	−0.13 **	−0.08 **	0.20
	Repair	−0.09	0.42 *	−0.32 **	−0.31	−0.18	−0.13 **	−0.01 *	0.24 **
Ecuador	Attention	0.05	0.07	0.02 **	0.04 **	0.10	−0.02	0.16	0.07 **
	Clarity	−0.15 **	0.22 **	−0.04	−0.22 **	−0.02 **	−0.01 **	0.19 **	0.16
	Repair	−0.21 **	0.30 **	−0.16 **	−0.24	−0.08 **	−0.04 **	0.13 **	0.09 **

* *p* < 0.05; ** *p* < 0.01; Note. PJ: personal justification; SB: shifting blame; LR: denial/lack of remorse.

**Table 5:** Descriptives and sex differences.

		Whole Sample					Spain					Argentina					Ecuador				
		*N*	*M*	*SD*	*t* (df)	Sig.	*N*	*M*	*SD*	*t* (df)	Sig.	*N*	*M*	*SD*	*t* (df)	Sig.	*N*	*M*	*SD*	*t* (df)	Sig.
ATTENTION	Total	1048	22.40	5.17			463	21.18	5.18			219	24.26	4.66			363	22.84	5.06		
	Men	537	21.86	5.16	−3.72 *** (1026)	0.00	363	21.59	5.09	3.23 (457)	0.001 ***	46	24.96	4.31	0.99 (209)	0.321	128	21.50	5.31	−3.84 (356)	0.000 **
	Women	491	23.04	5.05			96	19.69	5.33			165	24.22	4.45			230	23.60	4.75		
CLARITY	Total	1048	20.51	5.07			462	19.39	4.82			219	21	5.34			363	21.65	4.93		
	Men	540	20.18	5.06	−2.33 * (1030)	0.02	363	19.43	4.82	0.31 (457)	0.76	49	21.74	5.47	1.022 (214)	0.308	128	21.70	5.14	0.119 (356)	0.905
	Women	493	20.91	5.03			96	19.26	4.81			167	20.86	5.19			230	21.63	4.85		
REPAIR	Total	1048	20.51	5.66			462	18.56	5.47			220	21.01	5.48			363	22.69	5.14		
	Men	540	19.49	5.73	−2.12 *** (1031)	0.00	363	18.29	5.58	−2.03 (457)	0.043 *	49	19.98	5.42	−1.62 (214)	0.106	128	22.73	4.99	0.176 (356)	0.860
	Women	493	21.61	5.37			96	19.55	4.93			167	21.41	5.42			230	22.63	5.27		

Note: * *p* < 0.05; ** *p* < 0.01; *** *p* < 0.001.

## Data Availability

The data presented in this study are available on request from the corresponding author.
